# Comparison of perinatal outcomes of women with gestational diabetes mellitus according to type of treatment for glycemic control

**DOI:** 10.1016/j.jped.2024.03.016

**Published:** 2024-09-30

**Authors:** Pollyana Carvalho de Souza, Amanda Gabriela Araújo da Silva, Cristina Maria de Araújo Medeiros Santos, Luciana Araújo Cartaxo da Costa Santiago, Maria Elionês de Oliveira Araújo, Isabelle Lorena Barbosa de Lima, Karla Danielly da Silva Ribeiro

**Affiliations:** a Universidade Federal do Rio Grande do Norte, Maternidade Escola Januário Cicco, Programa de Residência Médica, Natal, RN, Brazil; b Universidade Federal do Rio Grande do Norte, Hospital Universitário Ana Bezerra, Natal, RN, Brazil; c Universidade Federal do Rio Grande do Norte, Maternidade Escola Januário Cicco, Programa de Pós-Graduação de Ciências Aplicadas à Saúde da Mulher, Natal, RN, Brazil; d Universidade Federal do Rio Grande do Norte, Maternidade Escola Januário Cicco, Natal, RN, Brazil; e Universidade Federal do Rio Grande do Norte, Departamento de Nutrição, Centro de Ciências da Saúde, Natal, RN, Brazil

**Keywords:** Gestational diabetes mellitus, Healthy lifestyle, Insulin, Perinatal results

## Abstract

**Objective:**

To compare the perinatal outcomes of women with Gestational Diabetes Mellitus (GDM), between pregnant treated only with lifestyle changes and pregnant treated with insulin and lifestyle changes.

**Methods:**

Prospective cohort study with follow-up of 64 women with GDM during the prenatal care and postpartum period until hospital discharge, divided into a control group (43) and an insulin group (21), with collection of sociodemographic, clinical, glycemic control and perinatal outcome data. Fetal macrosomia (≥ 4 Kg), or large-for-gestational-age newborns were considered the primary outcome of the research.

**Results:**

Pre-pregnancy BMI (31.2 ± 3.9 versus 28.8 ± 5.5), diastolic blood pressure (75 ± 8.7 versus 69 ± 6.9) and postprandial blood glucose (136.6 versus 115.4) ​​were higher in the insulin group, respectively. The control group had an average birth weight of 3058 g and an incidence of preterm birth of 11.6 %, while the insulin group had an average birth weight of 3203 g, with an incidence of preterm birth of 4.8 %. The majority of newborns had an adequate weight for their gestational age. Even all participants met glycemic goals, in the insulin group the Apgar score at the 5th minute and exclusive breastfeeding was lower, had 100 % of resuscitation cases, and a longer inpatient period.

**Conclusion:**

These data reinforce that even during prenatal care with lifestyle changes, newborns of women with GDM treated with insulin had worse outcomes, including clinical complications and less exclusive breastfeeding. It is important in prenatal care to identify neonates with risk for prevention and health promotion measures.

## Introduction

Gestational Diabetes Mellitus (GDM) is an intolerance to carbohydrates of varying severity, which begins during the current pregnancy, without having previously met the diagnostic criteria for diabetes mellitus (DM). In the Brazilian pregnant population, its prevalence varies from 3 % to 25 % depending on the population studied and the diagnostic criteria adopted.^1^ The primary objective in treatment is to achieve normoglycemia and, therefore, reduce the rates of adverse perinatal outcomes such as macrosomia, fetal death, and neonatal complications, such as birth injury, respiratory distress syndrome, and neonatal hypoglycemia.^2^

In a meta-analysis that included 14 Cochrane reviews, with a total of 17,984 women and 16,305 newborns, it was found that lifestyle changes (diet, physical activity and capillary glucose monitoring), when compared to usual prenatal care, was the only single intervention that showed a possible impact on the health of mothers and babies, with lower rates of large-for-gestational-age babies, although it increased rates of labor induction. Insulin treatment, in turn, was associated with an increase in hypertensive disorders during pregnancy when compared to oral therapy.^3^

In a retrospective cohort study that compared the perinatal outcomes of women with GDM in Belgium, insulin treatment did not prevent adverse events such as the rates of large-for-gestational-age newborns and these women have a higher risk profile, impaired beta-cell function and lower insulin sensitivity,^4^ but it is not yet known whether, even when treated with insulin therapy and lifestyle changes (diet and physical activity), there is a greater impact on perinatal outcomes, such as hospitalization, birth complications, and breastfeeding.

Therefore, considering that GDM corresponds to a condition that represents an important public health problem today and that more research is needed to understand the differences in other perinatal outcomes, such as birth complications and breastfeeding, between women treated with insulin therapy and lifestyle change and those treated with lifestyle change alone. In this study, the authors aim to compare the perinatal outcomes of pregnant women with GDM treated with lifestyle changes and pregnant women treated with insulin and lifestyle changes.

## Methods

This is a prospective cohort study evaluating the perinatal stages of pregnant women with GDM followed at the high-risk prenatal outpatient clinic of Maternidade Escola Januário Cicco (MEJC) in Natal/RN and evaluated via a specific form based on the diagnosis of GDM, until hospital discharge after birth, with the cohort lasting twenty-three weeks.

### Inclusion and exclusion criteria

Pregnant women diagnosed with GDM, with treatment instituted and initiated up to the 32nd week of pregnancy and undergoing high-risk prenatal care by the multidisciplinary team at the MEJC outpatient clinic, were included. Cases of multiple gestation, pregnant women with a previous diagnosis of Diabetes Mellitus, as well as pregnant women who smoke and have other comorbidities, such as Systemic Arterial Hypertension (chronic hypertension), heart disease, nephropathy, hepatopathy, thrombophilia, systemic lupus erythematosus and history of pancreatic disease, were excluded.

The diagnosis was made based on fasting blood glucose ≥ 92 (and < 126 mg/dL) at any gestational age or the oral glucose tolerance test 75 g (Fasting = 92–125 or 1 h postprandial ≥ 180 or 2 h postprandial = 153–199 mg/dL) carried out between 24 and 28 weeks of gestation.^1^

### Type of treatment

This research included pregnant women treated only with lifestyle changes – control group and pregnant women using insulin + lifestyle changes – insulin group. The lifestyle change was based on a normocaloric, high-protein diet and regular physical activity of at least 150 min per week of aerobic activity and/or resistance exercise. Insulin therapy, in turn, was performed with NPH and/or regular insulin available through the public health system in a personalized dose schedule according to the glycemic profile of each participant. The glycemic profile corresponds to the daily recording of 4 to 6 capillary blood glucose measurements. During the consultations, a food anamnesis and nutritional guidance were carried out according to the weight gain schedule during pregnancy.

**Figure 1:**
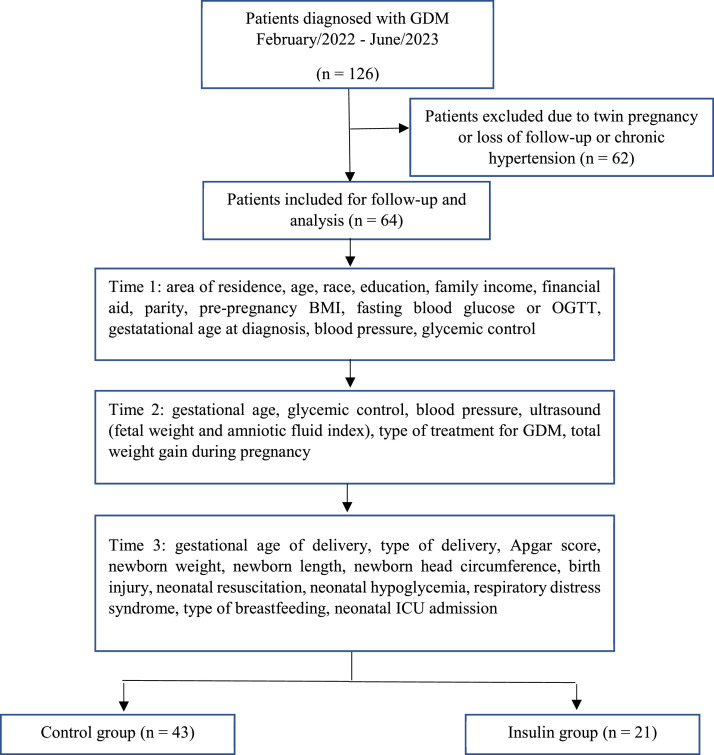
Study design flowchart. Follow-up Protocol.

Between February 2022 and June 2023, a total of 126 patients with GDM were recruited (Figure 1). Sixty-two patients were excluded due to twin pregnancy or had interrupted prenatal care or due to the presence of comorbidities, with chronic hypertension being the most prevalent. At the end of the cohort, the patients were allocated to their respective group, since during follow-up there was a need for medication intervention for some of them according to the result of the glycemic profile assessed at each consultation.

After acceptance and inclusion in the research, pregnant women were followed by an obstetrician, physical educator and nutritionist until delivery, in at least 5 consultations, with data collected from 3 moments for this research: time 1: until the 27th week of pregnancy; time 2: 28th – 36th week of pregnancy; and time 3: birth data and perinatal outcomes. Pregnant women in the Insulin group also underwent follow-up with an endocrinologist.

At such moments, data regarding glycemic control, weight gain, adherence to multidisciplinary monitoring, and physical activity were collected. An obstetric ultrasound was requested in the third trimester in order to track fetal repercussions of GDM through the assessment of fetal weight and amniotic fluid index (AFI). After birth, data regarding perinatal outcomes were collected.

### Sociodemographic data

Ethnicity was categorized as white and brown/black/indigenous, living area as urban and rural, family income as above and below the poverty line (0.5 minimum wage per capita), and education such as with and without completing high school.

### Clinical and glycemic control data

Data were collected regarding parity (number of pregnancies, births and abortions), pre-pregnancy body mass index (BMI), blood pressure, gestational age at the time of GDM diagnosis, weight gain during pregnancy, and follow-up with a nutritionist, physical educator and endocrinologist. Weight gain was considered adequate or inadequate considering the expectation of weight gain according to pre-pregnancy BMI, that is, low weight (BMI < 18.5) = 9.7 to 12.2 kg, adequate weight (BMI between 18.5 and 24.9) = 8 to 12 kg, overweight (BMI between 25 and 29.9) = 7 to 9 kg and obesity (BMI ≥ 30) = 5 to 7.2 kg.^5^

The monitored pregnant women brought a record of fasting and postprandial blood glucose levels to assess glycemic control (fasting < 95; 1 h postprandial < 140; 2 h postprandial < 120) at times 1 and 2, being considered adequate when it reached a minimum of 70 % of values ​​within normal limits, with the lowest possible frequency of hypoglycemia, according to current treatment protocols.^2^

### Perinatal outcomes

Perinatal outcomes were considered: gestational age at birth, birth weight, presence of fetal macrosomia (birth weight above 4 kg) or LGA newborn (large for gestational age, birth weight above the 90th percentile), occurrence of birth injury (shoulder dystocia, humerus or clavicle fracture, brachial plexus injury), need for neonatal resuscitation, exclusive breastfeeding and clinical complications in the newborn. These included episodes of neonatal hypoglycemia (blood glucose < 40 mg/dL), admission to the neonatal ICU, and respiratory distress syndrome. The presence of fetal macrosomia or LGA newborns was considered as primary outcome of the research, considering that GDM, when not well controlled, has an important impact on the weight of the fetus.

### Sample

Based on the proportions of 95.7 % (diet group) and 85 % (insulin group) of pregnant women with abnormal values ​​only for blood glucose measured after 2 h, the sample size of 188 patients was calculated considering a power of 80 % and an alpha error of 5 %.^6^ The G Power software version 3.1.9.7 was used. However, due to the number of women who met the study's exclusion criteria, sixty four pregnant women and their newborns were monitored, representing a sample power of 48 % considering the presented proportion of cases of inadequate glycemia between the control (non-exposed) and insulin (exposed) groups, and 95 % confidence interval.^7^

### Statistical analysis

In the final sample, data normality was assessed using the Kolmogorov-Smirnov test. For variables that did not present a normal distribution, the median and quartiles (1st and 3rd) were used. The descriptive analysis of variables that adhere to normal distribution was carried out using the mean and standard deviation. Categorical variables were expressed as absolute and relative frequencies.

The t tests for independent samples or Mann-Whitney were applied to evaluate differences in means. The Chi-square or Fisher tests were applied to analyze categorical variables. In situations where the table cells had expected frequencies lower than five, Fisher's exact test was applied.

### Ethical aspects of research

This research complies with the ethical and legal aspects of research involving human beings in accordance with Resolution n°. 466/2012, of the National Health Council. This research project was submitted to the Research Ethics Committee of the Hospital Universitário Onofre Lopes (HUOL/EBSERH), approved according to opinion no 5167,567 and CAAE n°.: 53,465,521.3.0000.5292 and data collection began only after approval. Collaborators were asked to voluntarily participate in the study by reading and signing the Free and Informed Consent Form, communicating the objective of the study and being guaranteed anonymity, confidentiality of information and the right to not participate, as well as to withdraw their consent at any time.

## Results

Table 1 shows the sociodemographic and economic characteristics of the participants. The average age of the participants was 30.2 years. In both groups, the majority of patients lived in urban areas, were of brown/black/indigenous race, had completed secondary education or higher and were socially vulnerable, where the majority had a per capita family income below the poverty line. However, there was no statistical difference between the groups in these analyzed variables.

**Table 1:** Sociodemographic and economic characterization of the participants, from FEBRUARY/22 to JUNE/23.

Variables	Control Group (*n* = 43)	Insulin group (*n* = 21)	Total (*n* = 64)	p-value
Age (mean, standard deviation)	29.5 (6.3)	31.8 (6.9)	30.2 (6.5)	0.252
	**n (%)**	**n (%)**	**n (%)**	
Residential Zone				
Urban area	36 (83.7)	18 (85.7)	54 (84.4)	0.575*
Countryside	7 (16.3)	3 (14.3)	10 (15.6)	
Race				
White	12 (27.9)	7 (33.3)	19 (29.7)	0.772
Brown/black/indigenous	31 (72.1)	14 (66.7)	45 (70.3)	
Education				
High school or graduation	34 (79.1)	17 (81.0)	51 (79.7)	0.570*
Junior high	9 (20.9)	4 (19.0)	13 (20.3)	
Family income				
Uninformed	5 (11.6)	1 (4.8)	6 (9.4)	0.371
Above the poverty line	15 (34.9)	11 (52.4)	26 (40.6)	
Below the poverty line	23 (53.5)	9 (42.8)	32 (50.0)	
Financial aid				
Uninformed	2 (4.6)	1 (4.8)	3 (4.7)	0.904
Yes	23 (53.5)	10 (47.6)	33 (51.6)	
No	18 (41.9)	10 (47.6)	28 (43.7)	

Chi-square test.

⁎ Fisher's exact test.

Table 2 shows clinical data of the pregnancy. The average number of pregnancies and abortions, as well as the gestational age (GA) at the time of GDM diagnosis were similar in both groups. Pre-pregnancy BMI and diastolic blood pressure were higher in the insulin group (*p* < 0.05).

**Table 2:** Clinical data on pregnancy, childbirth and postpartum, from February/2022 to June/2023.

Variables	Control Group (*n* = 43)	Insulin group (*n* = 21)	Total (*n* = 64)	p-value
Number of pregnancies (mean, standard deviation)	2.5 (1.3)	2.8 (1.8)	2.6 (1.5)	0.745
Number of abortions (mean, standard deviation)	0.4 (0.6)	0.5 (1.1)	0.4 (0.8)	0.806
Pre-pregnancy BMI in kg m^-1^² (mean, standard deviation)	28.8 (5.5)	31.2 (3.9)	29.6 (5.1)	**0.032**
Systolic blood pressure in mmHg (mean, standard deviation)	116 (11.8)	120 (14.7)	124 (15.9)	0.150
Diastolic blood pressure in mmHg (mean, standard deviation)	69 (6.9)	75 (8.7)	74 (12.2)	**0.001**
GA at diagnosis of GDM in weeks (mean, standard deviation)	15.6 (7.7)	15.7 (12.4)	15.7 (9.4)	0.448
Glycemic control at time 1				0.117
Uninformed, n (%)	4 (9.3)	1 (4.8)	5 (7.8)	
Adequate ⁎⁎, n (%)	36 (83.7)	10 (47.6)	46 (71.9)	
Inadequate, n (%)	3 (7.0)	10 (47.6)	13 (20.3)	
Glycemic control at time 2				
Uninformed, n (%)	2 (4.7)	0 (0.0)	2 (3.1)	
Adequate ⁎⁎, n (%)	40 (93.0)	18 (85.7)	58 (90.6)	
Inadequate, n (%)	1 (2.3)	3 (14.3)	4 (6.3)	
Weight gain during pregnancy⁎⁎⁎				
Adequate, n (%)	36 (83.7)	16 (76.2)	52 (81.2)	0.343*
Inadequate, n (%)	7 (16.3)	5 (23.8)	12 (18.8)	
Types of Insulin				–
One, n (%)	0	17 (81,0)	17 (81,0)	
Two, n (%)	0	4 (19,0)	4 (19,0)	
Number of insulin doses per day (mean, standard deviation)	0	2,24 (1,41)	2,24 (1,41)	0,382
Follow-up with a nutritionist				
Yes, n (%)	33 (76.7)	19 (90.5)	52 (81.3)	0.164*
No, n (%)	10 (23.3)	2 (9.5)	12 (18.7)	
Follow-up with a physical educator				
Yes, n (%)	17 (39.5)	10 (47.6)	27 (42.2)	0.539
No, n (%)	26 (60.5)	11 (52.4)	37 (57.8)	
Follow-up with an Endocrinologist				
Yes, n (%)	0 (0.0)	5 (23.8)	5 (7.8)	0.003*
No, n (%)	43 (100.0)	16 (76.2)	59 (92.2)	

Chi-square test.

⁎ Fisher's exact test.

⁎⁎ Adequate glycemic control: minimum of 70 % of values ​​within normal limits, with the lowest possible frequency of hypoglycemia.^2^

⁎⁎⁎ Weight gain was considered adequate or inadequate considering the expectation of weight gain according to pre-pregnancy BMI, that is, low weight (BMI < 18.5) = 9.7 to 12.2 kg, adequate weight (BMI between 18.5 and 24.9) = 8 to 12 kg, overweight (BMI between 25 and 29.9) = 7 to 9 kg and obesity (BMI ≥ 30) = 5 to 7.2 kg.^5^

Glycemic control was assessed at two stages, in the second and third trimester of pregnancy, and was found to be adequate in 71.9 % of women at time 1 and 90.6 % at time 2, with no statistical difference between the two groups. The majority showed adequate weight gain during pregnancy and were monitored by a multidisciplinary team. In turn, only 7.8 % were followed up with an endocrinologist, of which 100 % belonged to the insulin group (*p* = 0.003). In the insulin group, 81 % of patients used only one type of insulin (NPH or Regular) and 19 % of patients used two types (NPH and Regular) of insulin, with an average number of insulin doses of 2.24 to achieve adequate glycemic control.

Figure S2, available as supplementary material, shows glycemic profile data collected at times 1 and 2 of prenatal care. In glycemic self-monitoring, higher blood glucose values ​​were found in the insulin group in the post-breakfast measurement at time 1 (*p* = 0.008) and in the post-dinner measurement at both times (*p* < 0.01). However, it is worth highlighting that, in both groups and at both times, pregnant women had normal fasting and postprandial blood glucose levels.

Table 3 shows data regarding perinatal outcomes. The majority of newborns in both groups had birth weights classified as adequate for gestational age (AGA), and only one patient belonging to the insulin group had a pregnancy that resulted in fetal macrosomia (fetal weight ≥ 4 kg). Apgar score in the fifth minute was greater than 7 in both groups, however, it was lower in the insulin group compared to the control group (*p* = 0.011). The incidence of birth injury was only 1.6 % and occurred in a vaginal delivery. Regarding neonatal resuscitation, in most cases it was not necessary to perform it, however, 100 % of resuscitation cases occurred in the insulin group (*p* = 0.009). The majority of newborns in both groups did not develop neonatal hypoglycemia or neonatal respiratory distress syndrome.

**Table 3:** Perinatal outcomes of pregnant women with GDM followed in the study, February/2022 to June/2023.

Perinatal outcomes	Control Group (*n* = 43)	Insulin Group (*n* = 21)	Total (*n* = 64)	p-value
Gestational age at birth⁎⁎				
Full-term, n (%)	38 (88.4)	20 (95.2)	58 (90.6)	0.376
Preterm, n (%)	5 (11.6)	1 (4.8)	6 (9.4)	
Mode of delivery				
Vaginal, n (%)	13 (30.2)	4 (19.0)	17 (26.6)	0.262*
Cesarean section, n (%)	30 (69.8)	17 (81.0)	47 (73.4)	
AFI before delivery (mean, standard deviation)	12.7 (3.0)	12.5 (3.1)	12.6 (3.0)	0.479
Birth weight classification				
AGA, n (%)	37 (86.0)	19 (90.5)	56 (87.5)	0.604
LGA, n (%)	4 (9.3)	2 (9.5)	6 (9.4)	
SGA, n (%)	2 (4.7)	0 (0.0)	2 (3.1)	
Fetal macrossomia⁎⁎⁎				0.344
No, n (%)	43 (100.0)	20 (95.2)	63 (98.4)	
Yes, n (%)	0 (0.0)	1 (4.8)	1 (1.6)	
Birth weight in grams (mean, standard deviation)	3058 (407)	3203 (528)	3108 (453)	0.395
Length in cm (mean, standard deviation)	47.9 (1.8)	47.8 (2.6)	47.8 (2.1)	0.733
Head circumference in cm (mean, standard deviation)	34.4 (1.5)	34.8 (1.0)	34.5 (1.4)	0.200
Apgar score at the 5th minute (mean, standard deviation)	9 (0.3)	8 (0.5)	–	**0.011**
Birth injury				
No, n (%)	42 (97.7)	21 (100.0)	63 (98.4)	0.672*
Yes, n (%)	1 (2.3)	0 (0.0)	1 (1.6)	
Neonatal resuscitation				
No, n (%)	43 (100.0)	17 (81.0)	60 (93.8)	0.009*
Yes, n (%)	0 (0.0)	4 (19.0)	4 (6.2)	
Neonatal hypoglycemia				
No, n (%)	29 (67.4)	14 (66.7)	43 (67.2)	0.951
Yes, n (%)	14 (32.6)	7 (33.3)	21 (32.8)	
Neonatal respiratory distress syndrome				
No, n (%)	40 (93.0)	16 (76.2)	56 (87.5)	0.069*
Yes n (%)	3 (7.0)	5 (23.8)	8 (12.5)	
Breastfeeding				
Exclusive breastfeeding, n (%)	35 (81.4)	12 (57.1)	47 (73.4)	0.041*
Supplemented breastfeeding, n (%)	8 (18.6)	9 (42.9)	17 (26.6)	
Days of hospitalization in shared accommodation (mean, standard deviation)	3 (1.8)	3.7 (1.4)	3.2 (1.7)	**0.007**
Days of ICU stay (mean, standard deviation)	0.6 (2.2)	0.05 (0.2)	0.4 (1.8)	0.489

Chi-square test.

⁎ Fisher's exact test.

⁎⁎ Full-term: GA between ≥ 37 weeks; Preterm: GA < 37 weeks of gestation.

⁎⁎⁎ Birth weight above 4 kg.

Regarding breastfeeding, 73.4 % of all patients were exclusively breastfed, with the majority of them belonging to the control group (*p* = 0.041). Finally, the length of hospital stay was longer in the insulin group (3.7 days) compared to the control group (*p* = 0.007).

## Discussion

In this prospective observational cohort study of 64 women with singleton pregnancies affected by GDM and treated according to current protocols, with systematic screening and treatment according to the glycemic profile, the authors observed that the majority of pregnancies evolved with good glycemic control and adequate weight gain during prenatal care, full-term delivery and did not present with polyhydramnios. The majority of pregnant women had good adherence to lifestyle changes, with diet and physical activity and only pregnant women who needed to use insulin were referred to an endocrinologist, to better adjust insulin therapy.

The higher pre-pregnancy BMI in the Insulin group may have resulted in greater difficulty in glycemic control with diet/physical activity alone, generating the need for complementary treatment with insulin to achieve adequate glycemic control, which is already expected considering that overweight and obesity are important risk factors for GDM. This situation occurs due to greater insulin resistance, as was also observed in a cross-sectional study that evaluated 90 women with GDM at different stages of pregnancy.^8^ Higher postprandial glycemia values in the Insulin group also confirm the greater difficulty in glycemic control in this group, although both groups achieved the treatment target. In another study conducted in Vienna that evaluated 509 women with GDM, a greater need for medication intervention for glycemic control was also observed, where women with pre-gestational obesity required higher doses of insulin.^9^

The mean diastolic blood pressure (DBP) was also higher in the Insulin group, although still within the normal range (< 90 mmHg). In the HAPO study conducted in nine countries that included 23,316 non-diabetic participants, a relationship was observed between high BMI and a higher incidence of hypertensive disorders during pregnancy, including pre-eclampsia.^10^ The majority of newborns were classified as AGA, regardless of the type of treatment instituted, with an incidence of fetal macrosomia of only 1.6 %. Only 9 % of newborns were classified as LGA. This proportion was lower than that of a retrospective cohort study that evaluated 820 women with GDM in the Netherlands, which found 20 % of cases of LGA neonates.^6^

One of the main targets in the treatment of GDM is the reduction of LGA or macrosomic newborns, which consequently implies the reduction of negative outcomes such as shoulder dystocia, birth injury, neonatal hypoglycemia, neonatal resuscitation, and respiratory distress syndrome, in addition to possibly reducing the incidence of obesity and diabetes in adult life.^11^,^12^ Furthermore, it is known that LGA neonates are not always a consequence of GDM; factors such as overweight/obesity before pregnancy, maternal weight gain during pregnancy, and maternal age also influence this outcome.^13^,^14^

In a systematic review that evaluated 10 clinical trials in which data from 3317 patients with GDM who received treatment were included compared with 4407 patients with GDM who did not receive specific treatment, it showed that the intervention was associated with a significant reduction in the risk of macrosomia [OR 0, 3 (95 % CI 0.3–0.4)], LGA neonates [OR 0.4 (95 % CI 0.3–0.5)], shoulder dystocia [OR 0.3 (95 % CI 0. 2–0.6)], cesarean section [OR 0.8 (95 % CI 0.7–0.9)], pre-eclampsia [OR 0.4 (95 % CI 0.3–0.6)] and respiratory distress syndrome [OR 0.7 (95 % CI 0.5–0.9)]. However, in these studies no differences were found between the groups in the occurrence of newborns small for gestational age, hypoglycemia, hyperbilirubinemia, birth injury, admission to the neonatal ICU, and preterm birth, showing that intervention with the objective of achieving adequate glycemic control is safe, even in mild cases of GDM, with little altered blood glucose levels.^15^

The insulin group had a worse result on the Apgar score at the 5th minute, compared to the control group (8 versus 9; *p* = 0.011), as well as being responsible for 100 % of cases in which neonatal resuscitation was necessary (*p* = 0.009). The insulin group also had a longer stay in the hospital compared to the control group (*p* = 0.007). Regarding breastfeeding, 73.4 % of all participants did so exclusively, with the majority of them belonging to the control group (*p* = 0.041).

The insulin group had worse birth conditions with a greater need for neonatal resuscitation and a lower Apgar score at the 5th minute, as well as lower rates of exclusive breastfeeding and longer hospital stays. No statistically significant differences were found between the two groups in other perinatal outcomes, such as hypoglycemia, respiratory distress syndrome, birth injury, and admission to the neonatal ICU. Similarly, in a retrospective cohort study that evaluated 820 women with GDM in the Netherlands, no significant differences were observed in perinatal complications (mortality, birth injury, hyperbilirubinemia, hypoglycemia) as well as in the rates of LGA neonates, fetal macrosomia, Apgar < 7 on 5° minute, need for respiratory support, preterm birth and admission to neonatal ICU between the group treated with diet alone and the group treated with insulin.^6^

Compared to the control group, the insulin group presented a higher proportion of important perinatal outcomes, even in the face of multidisciplinary monitoring and protocols recommended by the Brazilian Diabetes Society, the Brazilian Federation of Gynecology and Obstetrics, and the Ministry of Health, which suggests the need for early diagnosis and multidisciplinary monitoring so that in the case of GDM there is no need for insulin treatment.

Despite the low number of women monitored and the use of the glycemic self-monitoring indicator, this work has potential as it followed women in social and biological vulnerability and managed to find differences in some outcomes. Such data confirm what is already known in the world literature about GDM and are even more important for the Brazilian population in the context of how GDM is managed, and how cesarean delivery is common.

These data reinforce that even during prenatal care with lifestyle changes, newborns of women with GDM treated with insulin had worse outcomes, including clinical complications and less exclusive breastfeeding. It is important to prenatal care to identify neonates with risk for prevention and health promotion measures, especially for the Brazilian population whose access to supplies for follow-up, as well as access to drug treatment, are difficult.

## Funding source

This study was financed in part by the Coordenação de Aperfeiçoamento de Pessoal de Nível Superior - Brasil (CAPES) - Finance Code 001.

## Conflicts of interest

The authors declare no conflicts of interest.

## Appendix Supplementary materials

Image, application 1

## Footnotes

Supplementary material associated with this article can be found in the online version at doi:10.1016/j.jped.2024.03.016.
