# Supplementary material for: Comparison of perinatal outcomes of women with gestational diabetes mellitus according to type of treatment for glycemic control
Source: J Pediatr (Rio J). 2024 Sep 30;101(2):179–86. doi: 10.1016/j.jped.2024.03.016 (PMC11889683; doi:10.1016/j.jped.2024.03.016)
Supplement: Supplementary file 1 [file mmc1.docx]

**JPED-D-23-00518 – Supplementary Material**

# supplementary material


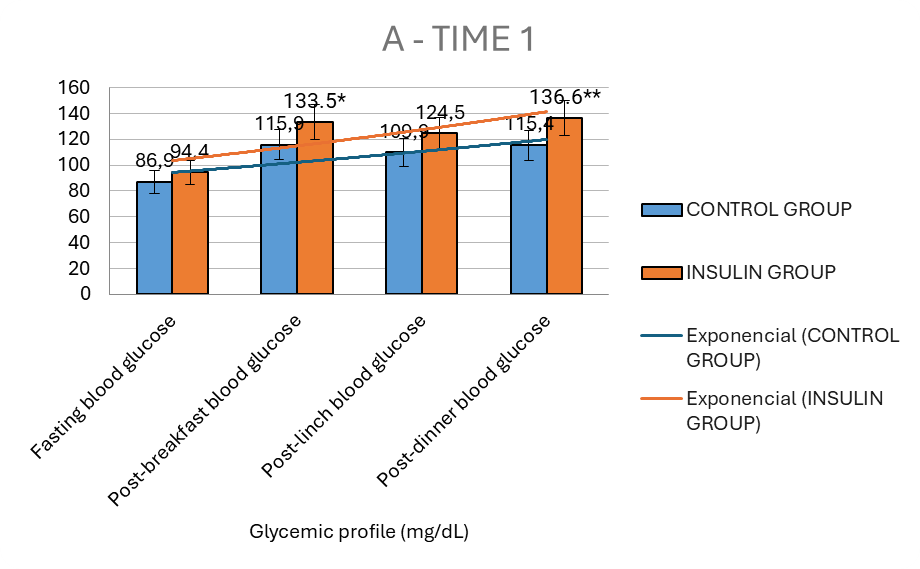

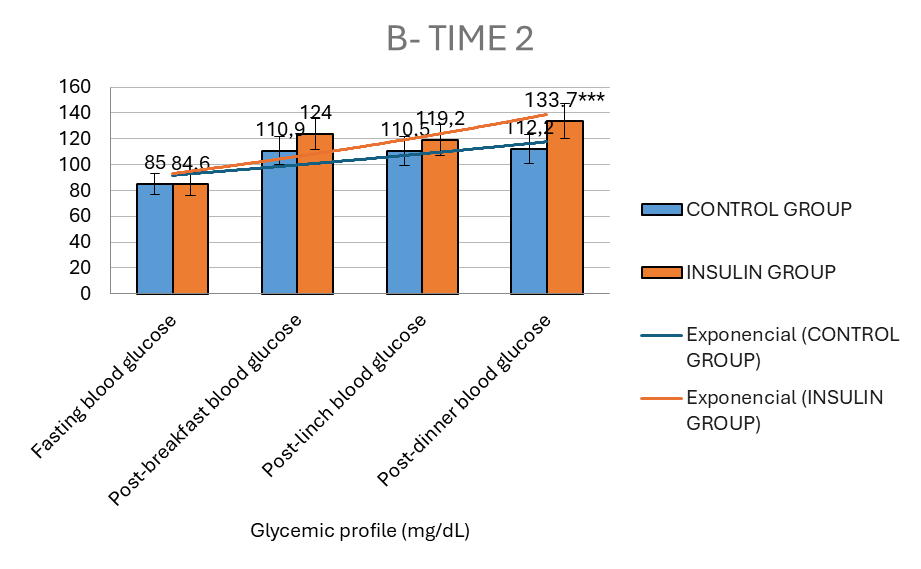


**Figure 2** Capillary blood glucose self-monitoring graph (mg/dL) at time 1 (A) and time 2 (B). * Significant difference between groups.
